# Short-course bortezomib-based retreatment for patients with multiple myeloma who had received bortezomib-thalidomide-dexamethasone (VTD) as an initial therapy: A single-center case series

**DOI:** 10.3892/etm.2014.1496

**Published:** 2014-01-23

**Authors:** JINGJUE MAO, FENG CHENG, HENG CHEN, JING WANG, XIN ZHOU, YUANQIANG JIANG, YUANXIN ZHU, HONGFENG GUO

**Affiliations:** Department of Hematology, Wuxi People’s Hospital, Nanjing Medical University, Wuxi, Jiangsu 214023, P.R. China

**Keywords:** bortezomib-based regimen, multiple myeloma, retreatment, resistance

## Abstract

Studies have shown that the bortezomib-based retreatment of patients with multiple myeloma (MM) may prolong control of the disease. The optimal duration of bortezomib-based retreatment in relapsed or refractory MM is unknown. The present retrospective study evaluated the efficacy and safety of short-course bortezomib-based retreatment in patients who had received bortezomib-thalidomide-dexamethasone (VTD) treatment for the initial therapy of newly diagnosed MM. The clinical records of 20 patients who had received short-course bortezomib-based retreatment in a single center were reviewed. Patients received a median of two cycles of bortezomib as the retreatment and the overall response rate was 90%. Six (30%), eight (40%) and four (20%) patients achieved a complete response (CR), a very good partial response and a partial response, respectively. Of the 10 patients who had achieved a CR during the initial VTD treatment, six experienced a repeat CR during the retreatment. The median duration of the response was nine months and the median time to progression was 10.5 months. The most common grade I and II adverse events were thrombocytopenia and neutropenia. The short-course bortezomib-based retreatment was well tolerated and the favorable response rates observed suggest that it may be an effective and convenient treatment option for certain patients, particularly elderly patients.

## Introduction

Multiple myeloma (MM) is a B-cell lymphoproliferative disorder that remains an aggressive and incurable disease. Despite the fact that novel targeted therapies have significantly improved the clinical outcome of MM patients in the frontline and recurrent settings, patients continue to experience disease progression and relapse, which requires the treatment to be changed (1). Retreatment with previously employed agents may be of benefit.

Bortezomib, a first-in-class proteasome inhibitor, has been shown to be effective in the treatment of relapsed or refractory MM (RRMM). Bortezomib-based regimens have also demonstrated enhanced activity, with high rates of complete response (CR) and very good partial response (VGPR), in patients with MM (2). In addition, a number of studies have provided evidence that the bortezomib retreatment of patients who have relapsed following bortezomib-containing therapy is feasible and effective, resulting in substantial clinical response rates (3–6). However, the available studies to date have not specifically addressed the optimal duration of bortezomib-based retreatment.

In a previous study, we administered a combination of bortezomib, thalidomide and dexamethasone (VTD) to patients with newly diagnosed MM (NDMM), and the overall response rate (ORR) was observed to be 91% (7). The present study concerns 20 of those patients who responded to the VTD therapy and then presented with progressive or relapsed disease and were retreated with bortezomib-based regimens. The results of the retreatment and the occurrence of adverse events (AEs) were evaluated.

## Patients and methods

This study involved the retrospective analysis of 65 patients who received VTD treatment as an initial therapy for NDMM. Of those patients, 20 who received bortezomib-based regimens as the salvage therapy for RRMM at some point during their MM disease course were included in the study group. The bortezomib-based regimens included VTD (7), a combination of bortezomib doxorubicin and dexamethasone (PAD), bortezomib-pegylated liposomal doxorubicin-dexamethasone (PLD), VTD plus allogeneic cytokine-induced killer cell therapy (8) and a combination of VTD with cisplatin, doxorubicin, cyclophosphamide and etoposide (PACE). The PAD regimen was composed of a three-week cycle of 1.3 mg/m^2^ bortezomib (Xian-Janssen Pharmaceutical Co., Ltd., Xian, Shanxi, China) on days 1, 4, 8, and 11, with 20 mg dexamethasone (Shandong Lukang Pharmaceutical Group Co., Ltd. Jining, Shangdong, China) on days 1–4 and 8–11 and 4.5 mg/m^2^ doxorubicin (Jiangsu Hansoh Pharmaceutical Co., Ltd., Lianyungang, Jiangsu, China) on days 1–4. The PLD regimen was composed of 20 mg/m^2^ pegylated liposomal doxorubicin (Shanghai Fudan-zhangjiang Bio-Pharmaceutical Co., Ltd., Shanghai, China) on day 1, with bortezomib and dexamethasone at the same dose and schedule as for the PAD regimen. The PACE regimen was composed of 10 mg/m^2^ cisplatin (Jiangsu Hansoh Pharmaceutical Co., Ltd.), 4.5 mg/m^2^ doxorubicin, 200 mg/day cyclophosphamide (Jiangsu Hengrui Medicine Co., Ltd., Lianyungang, Jiangsu, China) and 40 mg/m^2^ etoposide (Jiangsu Hengrui Medicine Co., Ltd.), all on days 1–4. It was recommended that patients were treated with two cycles of bortezomib following a confirmed CR or VGPR in the initial therapy (4). The main reasons for the short cycles of the bortezomib-based therapies were to avoid AEs and to overcome social factors such as the prohibitive cost of the treatment. All patients provided written informed consent. The study was conducted in accordance with the Declaration of Helsinki and with approval from the Hospital Review Board of Wuxi People’s Hospital, Wuxi, China.

The disease response following the initial therapy and salvage therapy was evaluated according to the International Myeloma Working Group criteria (9). Briefly, a CR was defined by a negative immunofixation test result for serum and urine, <5% plasma cells in the bone marrow and the disappearance of any soft-tissue plasmacytoma, if present at the baseline; a VGPR was defined as a reduction of ≥90% serum M-protein and urine M-protein levels <100 mg/24 h; and a partial response (PR) was defined as a ≥50% reduction of serum M-protein and reduction of 24-h M-protein by ≥90% or to <200 mg, and a ≥50% reduction in the size of the soft-tissue plasmacytomas. The ORR is the sum of the CR, VGPR and PR values.

Safety was assessed throughout the study, and AEs were graded according to the National Cancer Institute Common Toxicity Criteria (version 3.0) (10) and reported up to 30 days after the last dose of bortezomib.

Statistical analyses were performed using SPSS statistical software, version 13.0 (SPSS, Inc., Chicago, IL, USA). Time-to-event analyses were conducted using Kaplan-Meier methodology. The duration of response (DOR) was assessed only for patients achieving at least a PR and was calculated from the date of the first response to the date of progression. The time to progression (TTP) was from the date of the first administration of bortezomib to the date of progression.

## Results

### Patient characteristics

The baseline demographic and clinical characteristics of the 20 patients who received bortezomib-based retreatment are summarized in Table I. The median age of the patients at diagnosis was 63 years (range, 39–72 years). There were more men (n=14, 70%) than women and more patients identified with IgA myeloma (n=7, 35%) than with other myeloma types in this study. The karyotype analysis of the bone marrow of all the patients yielded normal results (data not shown).

### Initial VTD treatment and response

VTD treatment was used as the initial therapy in the 20 patients with MM. The study group received a median of two cycles (range, 2–4 cycles) of bortezomib treatment. Bortezomib (1.3 mg/m^2^) was administered on days 1, 4, 8 and 11 of each 21-day cycle. All patients achieved a PR or better with the initial VTD therapy; 10 patients (50%) achieved a CR, 8 (40%) achieved a VGPR and 2 (10%) achieved a PR. The median DOR was 18.2 months (range, 3.6–29.5 months) and the median TTP was 20 months (range, 6–32 months). Bortezomib was re-administered in the consecutive relapses.

### Interim anti-MM therapy

Between the initial VTD treatment and the bortezomib-based retreatment, the majority of the patients received MM-specific interim therapy. The interim therapies, as single agents or in combination, are summarized in Table I. A number of patients received more than one MM-specific therapy. For all the patients, the median time between the last dose of the initial bortezomib treatment and the first dose of an alternative antineoplastic therapy was 15 months (range, 4–25 months). The median time between the initial treatment and the retreatment with bortezomib (regardless of the interim therapies) was 20.5 months (range, 5–30.5 months).

### Bortezomib-based retreatment

**Figure 1:**
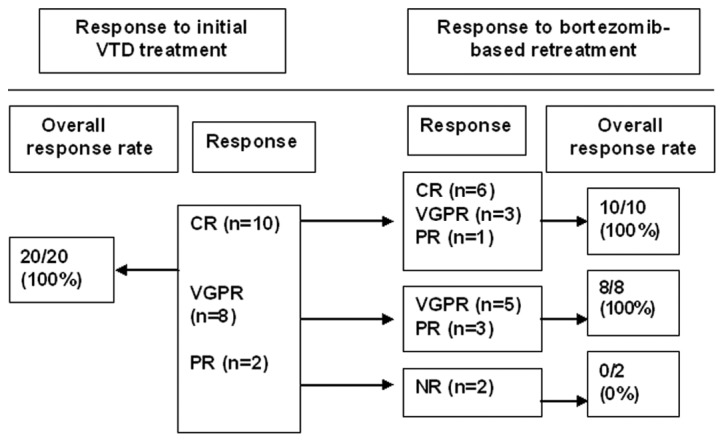
Response of the patients to the initial VTD treatment and the bortezomib-based retreatment. VTD, bortezomib, thalidomide and dexamethasone; CR, complete response; VGPR, very good partial response; PR, partial response; NR, no response.

The patients received a median of two therapies (range, 0–9 therapies) prior to the bortezomib-based retreatment (Table I) and a median of two cycles of bortezomib (range, 1–4 cycles) as the retreatment; 60% received two cycles. For the retreatment, 40% of the patients received the VTD regimen and 40% of the patients received the PAD regimen. The ORR to the bortezomib-based retreatment was 90%. Six (30%), eight (40%) and four (20%) patients achieved a CR, VGPR and PR, respectively. The association between the response to the initial VTD therapy and the response to the bortezomib-based retreatment is shown in Fig. 1. Of the 10 patients who achieved a CR during the initial VTD treatment, six experienced a repeat CR during the retreatment. Five of eight patients with an initial VGPR had a repeat VGPR, while the other three patients had a PR with the bortezomib-based retreatment. Of the two patients who achieved a PR during the initial VTD treatment, neither of them responded to the bortezomib-based retreatment. The median DOR to the bortezomib-based retreatment was 6.6 months (range, 0–11 months) and the median TTP was 8.7 months (range, 1–12.5 months).

**Figure 2:**
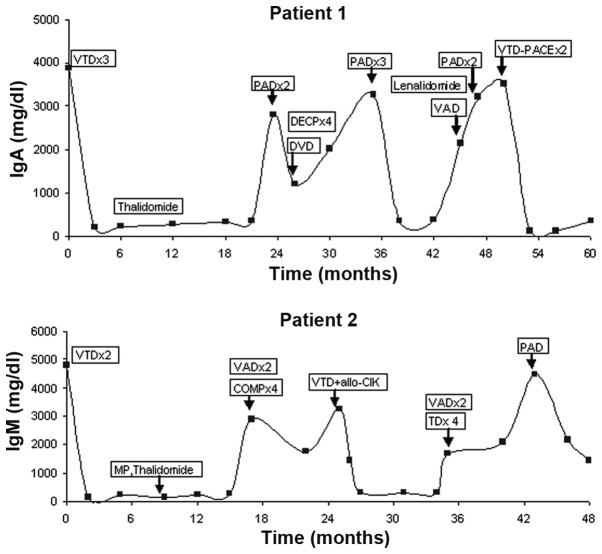
Diagram showing the M protein levels in relation to the events during the treatment of two of the patients. VTD, bortezomib, thalidomide and dexamethasone; PAD, bortezomib, doxorubicin and dexamethasone; DVD, doxorubicin, vincristine and dexamethasone; DECP, dexamethasone, etoposide, cyclophosphamide and cisplatin; VAD, vincristine, adriamycin and dexamethasone; PACE, cisplatin, doxorubicin, cyclophosphamide and etoposide; MP, melphalan and prednisone; COMP, cyclophosphamide, vincristine, melphalan and prednisone; allo-CIK, allogenic cytokine-induced killer; TD, thalidomide and dexamethasone.

Patient 1 was treated four times with bortezomib, with a 23.5-month break between the first and second, a 11.5-month interval between the second and third, and a 12-month break between the third and fourth therapy courses. The first, third and fourth treatments resulted in CRs which lasted 21.5, 10 and 7 months, respectively, and the second treatment resulted in a PR which lasted 11 months. Patient 2 was treated three times with bortezomib, with a 25-month break between the first and second and a 21-month interval between the second and third therapy courses. The initial treatment resulted in a CR which lasted 14.7 months, the second treatment resulted in a VGPR which lasted 8 months and the third treatment resulted in a PR which lasted 6 months. Fig. 2 shows the M protein levels (IgA normal levels, 69–382 mg/dl; IgM normal levels, 63–277 mg/dl) in relation to the events during the treatment of patient 1 and 2.

### Safety and tolerability

The AEs during the bortezomib-based retreatment are shown in Table II. During the retreatment, the most common AEs were thrombocytopenia and neutropenia. The majority of the AEs were grade I or II. Interruption of the therapy was required in three patients; discontinuation was due to pulmonary infection, diarrhea and neutropenia. Two patients passed away during the retreatment period: One succumbed due to sepsis and one due to disease progression. All mortalities were considered to not be associated with bortezomib.

## Discussion

Over the past 10 years, the introduction of novel agents such as thalidomide, bortezomib and lenalidomide has markedly changed the treatment of patients with NDMM or RRMM (11). Although the single agent activity of these compounds has been reported in MM, their major impact in the management of the disease is observed through combination regimens. Preclinical studies have demonstrated that bortezomib enhances the antiproliferative and proapoptotic activity of cytotoxic agents such as melphalan and doxorubicin against myeloma cells (12). Preclinical observations have also identified synergistic anti-myeloma activity of bortezomib when combined with an immunomodulatory drug (13). The results of a number of clinical trials have shown that bortezomib-based regimens are an effective treatment against NDMM and RRMM (14). Briefly, bortezomib-based regimens show potential for increasing the depth (CR/VGPR) and durability of responses, overwhelming possible resistance and improving survival.

In patients with RRMM, there is an urgent requirement to optimize treatment regimens to extend the duration of survival. The duration of the first remission and the timing of the relapse are key determinants for the treatment strategy at the relapse. The National Comprehensive Cancer Network clinical practice guidelines state that patients with MM who are not refractory to the initial therapy (relapse >6 months after completion of the previous therapy) may be retreated with the same regimen (15). Bortezomib is a weak substrate for multi-drug resistance efflux pumps and has the potential to avoid resistance. In a phase III VISTA trial, it was shown that patients relapsing following bortezomib-based therapy were not intrinsically more resistant to subsequent therapies compared with those relapsing following traditional chemotherapy with melphalan and prednisone, and they were successfully treated with subsequent bortezomib-based therapies (16,17). The effectiveness of the retreatment with bortezomib may be a result of certain patients experiencing a clinical response to the initial treatment and therefore remaining sensitive to the bortezomib-based retreatment. It is also possible that the addition of other agents to the bortezomib-based retreatment may have contributed to the responses observed.

The present retrospective case series represents the first review of bortezomib-based retreatment exclusively in the Chinese clinical practice setting, in patients who had responded to VTD treatment for the initial therapy of NDMM. The results presented demonstrate the efficacy of short-course bortezomib-based retreatment, with an ORR of 90%. The response to the bortezomib-based retreatment was most notable in the patients who had exhibited a good response to the initial VTD therapy. Crucially, in one of the heavily pretreated relapsed MM patients, a triple response (PR, VGCR and CR) following the bortezomib-based retreatment was achieved, and in another patient a double response (VGPR and PR) was achieved. These findings suggest that the treatment-free interval of >6 months following the initial bortezomib therapy was associated with a superior response rate and may be predictive of the efficacy of bortezomib-based retreatment. Although the number of patients studied is small, the data indicate a trend towards a greater sensitivity to short-course bortezomib-based retreatment than during initial treatment in patients who had responded to VTD treatment for the initial therapy of NDMM, even in heavily pre-treated patients.

The toxicities reported in the present study were manageable and generally predictable. It is possible to manage the hematological toxicity of the treatment using dose modifications and/or growth factor support. Severe myelosuppression was uncommon. Grade III or IV neutropenia, related febrile neutropenia and sepsis were rare. The thrombocytopenia observed was transient and not associated with serious bleeding complications. The treatment-induced peripheral neuropathy (PN) was lower during the bortezomib-based retreatment. The short duration of the therapy and the dose modifications in the study may have minimized the cumulative PN arising from the bortezomib-based retreatment. These findings suggest that retreatment with short-course bortezomib-based combination regimens may be a well-tolerated and effective therapeutic option for patients who respond to the initial VTD therapy and that it is possible to re-use bortezomib in subsequent lines of therapy without resistance.

The responses to the bortezomib-based retreatment in the present study were rapid, with 60% of responders achieving their best response by the end of cycle two. However, the bortezomib-based retreatment was associated with a shorter DOR and TTP than the initial VTD treatment was, which is commonly observed with subsequent lines of therapy (3–5,16) due to the progressive disease course. The higher CR rates in the VISTA and phase II studies may be due to the prolonged courses of bortezomib-based retreatment improving the quality of the response in a proportion of the patients (3–5,16). However, prolonged therapy may be associated with continued toxicity in certain patients, as well as the inconvenience of having to attend frequent hospital appointments for a long period of time. Therefore, the findings of the present study may indicate highly active, tolerable and convenient treatment options with a reduced treatment burden for certain patients, particularly for elderly patients.

Clearly, the higher response rate is a significant observation. However, this study has limitations due to the small sample size and retrospective design; a longer follow-up and prospective clinical trials are required to validate these initial observations. This study is likely to contribute to the identification of the optimal sequence of treatments for individual patients while balancing efficacy and toxicity.

## Figures and Tables

**Table I:** Baseline demographic and clinical patient characteristics.

Characteristics	Patients (n=20)
Median age at diagnosis, years (range)	63 (39–72)
Males, n (%)	14 (70)
Myeloma type, n	
IgG	9
IgA	7
IgM	1
Light chain	2
IgD	1
Median time from diagnosis to bortezomib-based retreatment, months (range)	20.5 (5–30.5)
Therapies prior to bortezomib-based retreatment	
Median number of prior lines of therapy including bortezomib, n (range)	4 (2–11)
Received in prior regimen (other than bortezomib), n (%)	
Thalidomide and dexamethasone	6 (30)
Melphalan and prednisone	6 (30)
Vincristine, doxorubicin and dexamethasone (VAD)	14 (70)
α-interferons	2 (10)
Thalidomide	20 (100)
Bone marrow transplant	1 (5)
Cyclophosphamide, vincristine, melphalan and prednisone (COMP)	6 (30)
Other	2 (10)

**Table II:** Adverse events during the bortezomib-based retreatment.

Adverse event	Total patients	Severity (grade)			

		I	II	III	IV
Diarrhea	2	1		1	0
Thrombocytopenia	10	4	4	2	0
Neutropenia	8	2	4	2	0
Weakness	2	2	0	0	0
Pulmonary infection	1	0	0	1	0
Peripheral neuropathy	2	1	1	0	0
Herpes zoster	2	1	1	0	0

All data presented as number of patients. AEs, adverse events.
